# Biopsychosocial Health Care Needs at the Emergency Room: Challenge of Complexity

**DOI:** 10.1371/journal.pone.0041775

**Published:** 2012-08-28

**Authors:** Franziska Matzer, Ursula V. Wisiak, Monika Graninger, Wolfgang Söllner, Hans Peter Stilling, Monika Glawischnig-Goschnik, Andreas Lueger, Christian Fazekas

**Affiliations:** 1 Department of Medical Psychology and Psychotherapy, Medical University of Graz, Graz, Austria; 2 Department of Psychosomatics and Psychotherapy, General Hospital Nuremberg, Nuremberg, Germany; 3 Department of Internal Medicine, Medical University of Graz, Graz, Austria; Federal University of Rio de Janeiro, Brazil

## Abstract

**Background:**

In an emergency room of internal medicine, triage and treatment of patients deserve first priority. However, biopsychosocial case complexity may also affect patient health outcome but has not yet been explored in this setting. Therefore, the aims of the study are (1) to estimate prevalence rates of complex patients in the emergency room (ER), (2) to describe biopsychosocial complexity in this population and (3) to evaluate possible correlations between patient profiles regarding case complexity and further clinical treatment.

**Methods:**

During a study period of one week, all patients of an emergency room of internal medicine who were triaged to Manchester levels three to five were invited to participate in the study. Biopsychosocial case complexity was assessed by the INTERMED method. Psychosocial interventions were evaluated based on all documented interventions and recommendations given at the emergency room and during inpatient treatment.

**Results:**

Study participants consisted of 167 patients with a subgroup of 19% (n = 32) receiving subsequent inpatient-treatment at the department. High biopsychosocial case complexity was found in 12% (n = 20) of the total sample (INTERMED score >20). This finding was paralleled by a cluster analysis suggesting three clusters with one highly complex patient group of 14%. These highly complex patients differed significantly from the other clusters as they had visited the emergency room more often within the last year and lived alone more frequently. In addition, admission rates were highest in this group. During ER treatment and subsequent inpatient treatment, 21% of highly complex patients received interventions addressing psychosocial factors as compared to 6% and 7%, respectively, in the other clusters.

**Conclusions:**

A standardized screening of biopsychosocial case complexity among ‘frequent utilizers’ of the ER would be helpful to detect specific multidisciplinary health care needs among this particularly burdened patient group.

## Introduction

Biopsychosocial case complexity implies concurrence of somatic disease and psychiatric disorders or other psychosocial or health care-related factors that contribute to a high degree of overall disease burden. As measured by the INTERMED method, complexity indicates a need for multidisciplinary and integrated treatment. This has been demonstrated for different patient groups [1]–[5]. Compelling evidence suggests that biopsychosocial case complexity influences patient health outcomes [6], [7], health care use [8], patient management [9], [10] and health care expenditure [11]. With the INTERMED method, a validated clinimetric instrument, biological, psychological, social and health care-related aspects of disease are rated resulting in a patient profile in these four domains. All four domains contribute to an integral assessment of patient care needs as they jointly influence case complexity, thus suggesting this construct to be one-dimensional [12], [13]. While prevalence rates of case complexity seem to vary widely between medical fields, e. g. 11% in patients with multiples sclerosis [14] and 24% in pneumology inpatients [15], cumulative data have demonstrated that high scores on the INTERMED predict negative outcomes such as long hospital stay, poor results in patients with diabetes and poor discharge status in patients admitted to internal medicine [3], [6], [15].

In an emergency room (ER) of internal medicine, triage of patients and patient management are crucial and among several existing triage systems, the Manchester Triage System is the most commonly used in German-speaking countries [16], [17]. Patient management should be based on an integral biopsychosocial assessment of patient care needs as soon as possible, particularly in patients who are not triaged as medically very urgent and who belong to the group of frequent utilizers of this health care segment [18], [19]. The importance of psychosocial factors and psychiatric comorbidity in patient care in an emergency room of internal medicine has been widely established, for example in patients with psychological problems and panic disorders who present non-cardiac chest pain [20], [21] or for patients at risk for emergency care utilization due to depressive or addictive disorders [22], [23]. Nevertheless, the INTERMED method has not been applied in an emergency room and biopsychosocial case complexity has not been investigated. Therefore, the aims of the study are (1) to estimate prevalence rates of complex patients in the emergency room (ER), (2) to describe biopsychosocial complexity in this population and (3) to evaluate possible correlations between patient profiles regarding case complexity and further clinical treatment. We assumed that high case complexity would be associated with (1) more prior visits to the emergency room, (2) more frequent admission to the hospital after the ER visit, (3) more specific diagnostic procedures in the current ER visit and (4) more psychosocial interventions concerning multidisciplinary health care needs in the ER and during admission. For this reason we also assessed all documented biopsychosocial interventions at the ER and all documented psychosocial interventions during subsequent inpatient treatment.

## Methods

### Study design and participants

The study was approved by the ethics committee of the Medical University of Graz (IRB00002556).

Participants were recruited from the university's emergency room for internal medicine. During one week in March 2010 (seven days, 24 hours), all patients visiting the ER were documented by the ER triage nurses and screened in terms of inclusion criteria: patients at the age of ≥18 years, rated in triage categories *urgent* to *non urgent* (categories three to five), and willing to participate. Exclusion criteria were an *immediate* or *very urgent* Manchester triage rating (categories one and two), cognitive impairment and/or insufficient knowledge of German. Using these criteria, 167 patients participated in the study (Table 1).

**Table 1:** Inclusion process.

Patients within one week		325
Inclusion		167
Exclusion		158
	Manchester Triage 1/2	63
	Cognitive impairment	28
	Inadequate knowledge of German language	10
	Did not want to participate	31
	Did not want to participate because of health problems	18
	Logistic matters	7
	Test aborted	1

Patients who met the inclusion criteria were informed about the study and invited to participate. If they agreed, written informed consent was obtained. Afterwards, a sociodemographic data sheet was completed with support of a study nurse. After a standard physical examination following routine ER procedure, the waiting period for laboratory parameters and/or the discharge letter was utilised to conduct the INTERMED interviews. Two study nurses logistically supported this course of action to guarantee the smooth integration of the interviews over the course of standard medical treatment at the ER. Sociodemographic characteristics of the sample are presented in Table 2.

**Table 2:** Sociodemographic characteristics of the sample.

Sociodemographic characteristics		n (%)
Sex	Male	76 (45.5%)
	Female	91 (54.5%)
Age (years)	Mean (SD)	50.7 (21.2)
	<40	58 (34.7%)
	40–59	49 (29.3%)
	>59	60 (35.9%)
Nationality	Austrian	147 (88.0%)
	Other	20 (12.0%)
Situation of living	Alone	41 (24.6%)
	Alone with kid(s)	10 (6.0%)
	In institution	3 (1.8%)
	In flat-sharing community	6 (3.6%)
	With parents	8 (4.8%)
	With partner	66 (39.5%)
	With partner and kid(s)	33 (19.8%)
Marital status	Unmarried	55 (32.9%)
	Married	73 (43.7%)
	Divorced	20 (12.0%)
	Widowed	18 (10.8%)
	Unknown	1 (0.6%)
Job situation	Unemployed	9 (5.4%)
	In education/schooling	11 (6.6%)
	Home maker	4 (2.4%)
	Marginal employment	4 (2.4%)
	Part-time work	7 (4.2%)
	Full-time work	60 (35.9%)
	Retired	65 (38.9%)
Level of education	Primary education	15 (9.0%)
	Secondary education 1^st^ stage	23 (13.8%)
	Secondary education 2^nd^ stage	64 (38.3%)
	Post secondary education	37 (22.2%)
	Higher education (university)	28 (16.8%)

The interview team consisted of 16 MDs, psychologists and psychotherapists from the Department of Medical Psychology and Psychotherapy at the Medical University of Graz. As the interviews also took place during peak time, up to four interviewers had to be present at the ER, and it was necessary to provide a large team of interviewers in order to secure an around-the-clock investigation. INTERMED training was conducted by one of the authors (W.S.) who developed the German version of this instrument in collaboration with the INTERMED work group. Group ratings and double ratings with the trainer took place, until all trainees were officially certified as INTERMED interviewers.

After patients had received ER treatment, additional information and documents were obtained via the electronic documentation system MEDOCS. For all participants the number of prior ER visits within the last year was registered. All current ER discharge letters were analyzed regarding documented biopsychosocial interventions and recommendations for further treatment. In addition, for all patients with subsequent inpatient treatment in a department at the Medical University of Graz, discharge letters were evaluated concerning all documented psychosocial interventions during their stay and corresponding recommendations for further treatment.

### Measurements

**Figure 1:**
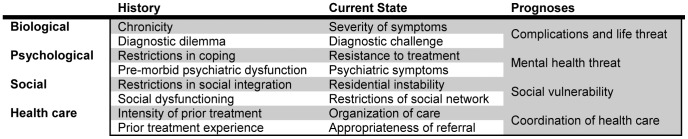
Variables of the INTERMED grid (adapted from de Jonge et al., 2005 [**7**]).

The INTERMED is an observer-rated and semi-structured clinical interview based on the structured taking of a medical history. It assesses biopsychosocial case complexity. Various studies and evaluations in different clinical settings have proven its reliability and validity [7], [24]. Biopsychosocial case complexity refers to four domains: biological, psychological, social, and health care. These domains are evaluated with regard to time (history, current state, and prognosis), resulting in 12 cells of information with all together 20 pertinent variables (*Figure 1*). All variables are rated with a score ranging from 0 to 3, with a higher score indicating a higher degree of case complexity. The scores can be summed up to a domain score (maximum score of 15 for each of the four domains) and a total score of biopsychosocial case complexity (maximum score of 60). A cut-off score of >20 indicates highly complex patients. The duration of the INTERMED interview is about 20 minutes.

### Evaluation of discharge letters

ER discharge letters were evaluated by two MDs, an internist and a psychiatrist, according to the following questions: current main diagnosis, number of chronic diseases, number and type of diagnostic interventions, drug intake at the ER, patient management after ER discharge, and recommendations for further treatment. For patients with subsequent admission, the length of stay, type and number of documented psychosocial interventions during the stay, and recommendations for further treatment were additionally collected.

### Data analysis

Statistical analysis was conducted with SPSS 16.0. Descriptive data are presented by absolute frequencies and percentages or by means and standard deviations.

As this was the first time the INTERMED had been applied in an emergency room, we aimed to gain comprehensive insight into biopsychosocial complexity in this setting. For this reason case complexity was calculated in two different ways. First, the well-established INTERMED cut-off score of >20 was used to assess prevalence rates of high biopsychosocial case complexity. Second, due to the exploratory nature of this study, we aimed at gaining a more differentiated picture of complexity in this study cohort than a single cut-off score could provide. Thus, a hierarchical cluster analysis using Ward's method based on the 20 INTERMED variables was conducted. This nonparametric method allows one to identify subjects that are similar on the basis of Euclidean distances. A screen plot analysis revealed two or three clusters according to the contingent coefficient. We decided to choose the three-cluster-solution, as it facilitates a more differentiated description of the sample (within the two-cluster-solution, clusters one and two would have overlapped).

To facilitate comparisons between the three clusters, some sub-categories of categorical demographic variables were combined in these analyses. Group comparisons among continuous variables were conducted using ANOVAs for independent samples or an independent sample t-test. For comparisons between categorical variables a Pearson's χ^2^ test was used. A probability p-value of less than 0.05 was considered significant.

## Results

### Prevalence of case complexity in the emergency room setting

General characteristics of the sample in relation to the ER are shown in Table 3. ER visits during the night are common, as one third were treated between 8 pm and 8 am. About 25% of study participants had had one or more prior visits to the ER during the last year.

**Table 3:** ER-related data of the sample.

ER-related data		n (%)
Context of referral to ER	General practitioner	30 (18.0%)
	Ambulance	60 (35.9%)
	Self-admission	74 (44.3%)
	Other	3 (1.8%)
Time of ER visit	Daytime (8 am–8 pm)	108 (64.7%)
	Night time (8 pm–8 am)	59 (35.3%)
Manchester Triage category	3 (urgent)	66 (39.5%)
	4 (standard)	95 (56.9%)
	5 (non-urgent)	6 (3.6%)
Presenting symptoms	Vomitus/diarrhoea	32 (19.2%)
	Collapse/circulation	29 (17.4%)
	Abdominal pain	26 (15.6%)
	Breathing difficulties	20 (12.0%)
	Peripheral pain	17 (10.2%)
	Chest pain	17 (10.2%)
	Infections/fever	13 (7.8%)
	Blood pressure	7 (4.2%)
	Worsening general condition	3 (1.8%)
	Pelvic pain	3 (1.8%)
Prior visits to ER in last year	0	126 (75.4%)
	1	24 (14.4%)
	2	11 (6.6%)
	≥3	6 (3.6%)

Based on the INTERMED cut-off score, 20 patients (12%) were identified as highly complex among study participants.

To explore the full range of biopsychosocial case complexity in a more differentiated way, a cluster analysis was calculated that revealed three clusters of ER patients. As expected, the cluster comprising the most complex patients resembled the subgroup with an INTERMED total score of >20. It encompassed 24 patients (14,4%).

### Characteristics of clusters of case complexity

**Figure 2:**
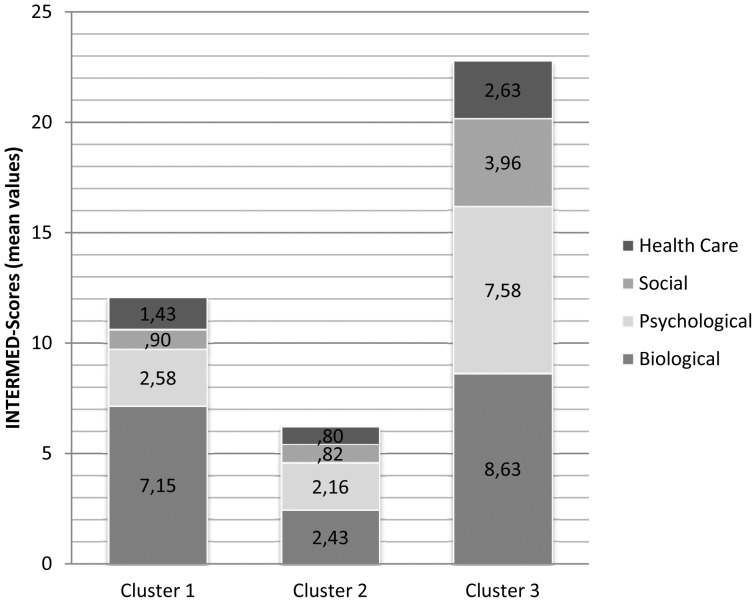
Description of the three clusters of patients by INTERMED domain scores summing up to total scores.

In Figure 2 the clusters' mean scores in the four INTERMED domains are presented, summing up to the total INTERMED score of each cluster. Cluster one (n = 92, 55.1%) consists of more than half of the sample who have a high somatic score, but low psychosocial case complexity. Cluster two (n = 51, 30.5%), approximately one third of the sample, can be described as a patient group with low INTERMED scores concerning all four domains. As can be seen in Table 4, patients in cluster two are younger than the others, have obtained higher education and tend to visit the ER more often at night. They have few chronic diseases and seldom need inpatient treatment.

**Table 4:** Discriminating factors between INTERMED clusters.

		Total	Cluster 1	Cluster 2	Cluster 3	Statistics
Age (years)	Mean (SD)	50.7 (21.2)	57.6 (20.6)	35.6 (14.8)	56.5 (19.4)	F = 23.76, p<0.001** χ2 = 37.94, p<0.001**
	<40	58 (34.7%)	21 (22.8%)	34 (66.7%)	3 (12.5%)	
	40–59	49 (29.3%)	27 (29.3%)	12 (23.5%)	10 (41.7%)	
	>59	60 (35.9%)	44 (47.8%)	5 (9.8%)	11 (45.8%)	
Situation of living	Alone	41 (24.6%)	20 (21.7%)	10 (19.6%)	11 (45.8%)	?2 = 6.93, p = 0.031*
	Other	126 (75.4%)	72 (78.3%)	41 (80.4%)	13 (54.2%)	
Level of education	Primary/secondary	102 (61.1%)	62 (67.4%)	24 (47.1%)	16 (66.7%)	?2 = 6.07, p = 0.048*
	Post secondary/higher	65 (38.9%)	30 (32.6%)	27 (52.9%)	8 (33.3%)	
ER visits last year	No	126 (75.4%)	68 (73.9%)	44 (86.3%)	14 (58.3%)	?2 = 7.14, p = 0.028*
	Yes	41 (24.6%)	24 (26.1%)	7 (13.7%)	10 (41.7%)	
Time of ER visit	Daytime (8 am–8 pm)	108 (64.7%)	63 (68.5%)	26 (51.0%)	19 (79.2%)	?2 = 6.98, p = 0.031*
	Night-time (8 pm–8 am)	59 (35.3%)	29 (31.5%)	25 (49.0%)	5 (20.8%)	
Chronic diseases	No	61 (36.7%)	19 (20.9%)	36 (70.6%)	6 (25.0%)	?2 = 36.41, p<0.001**
	Yes	105 (63.3%)	72 (79.1%)	5 (29.4%)	18 (75.0%)	
Admission	No	127 (76.0%)	64 (69.6%)	48 (94.1%)	15 (62.5%)	?2 = 13.68, p = 0.001**
	Yes	40 (24.0%)	28 (30.4%)	3 (5.9%)	9 (37.5%)	

* p<0.05,

** p<0.001.

Cluster three (n = 24, 14.4%) refers to a highly complex patient group with higher scores in all four INTERMED domains. Cluster three resembles cluster one according to the somatic INTERMED score, age and a high number of chronic diseases. However, in contrast to cluster one patients, cluster three patients live alone more frequently (45.8% vs. 21.7%). As expected, they have visited the ER more often than patients of cluster one (41.7% vs. 26.1%). In addition, and in accordance with our assumption, patients in this cluster seem to need inpatient treatment most frequently.

### Case complexity and clinical treatment

As presented in Table 5, the evaluation of ER discharge letters focused on the diagnosis given in the ER, number of chronic diseases, diagnostic and therapeutic interventions, application of drugs, and documented recommendations for further patient management. During the ER visit, six patients (3.6%) received psychosocial interventions (psychopharmacological therapy) or recommendations for further treatment by a psychiatrist, psychologist or psychotherapist.

**Table 5:** Evaluation of ER discharge letters (n = 167).

Evaluation of ER discharge letters		n (%)
Current diagnosis	gastrointestinal	48 (28.7%)
	cardiovascular	45 (26.9%)
	pulmonary	24 (14.4%)
	psychosomatic	11 (6.6%)
	musculoskeletal	10 (6.0%)
	other	28 (16.8%)
	missing	1 (0.6%)
Chronic diseases	0	61 (36.5%)
	1	37 (22.2%)
	2 or more	68 (40.7%)
	missing	1 (0.6%)
Diagnostic interventions at ER	Standard (physical examination, lab, ECG)	79 (47.3%)
	Standard+ultrasonic, X-ray, puncture, expert consultation, MRT and/or CT	87 (52.1%)
	missing	1 (0.6%)
Application of drugs	General drugs (oral, subcutaneous, intravenous)	87 (52.2%)
	Psychotropic drugs	1 (0.6%)
	Analgesic drugs (intravenous)	14 (8.4%)
Patient management after ER discharge	No admission or further diagnostics at policlinic	119 (71.3%)
	Further diagnostics at policlinic	8 (4.8%)
	Admission	40 (24%)
Admission to	Department of Internal Medicine	29 (72.5%)
	Other department	3 (7.5%)
	External hospital	8 (20%)
Recommendations for further treatment (n = 127)*	No recommendations	48 (37.8%)
	General practitioner	25 (19.7%)
	Medical specialist	59 (46.5%)
	Psychiatrist/psychologist/psychotherapist	5 (3.9%)

ER = Emergency Room, ECG = Electrocardiogram, MRT = magnetic resonance tomography, CT = computer tomography.

* Documented recommendations refer to a subgroup of patients without admission after ER treatment.

In contrast to the hypothesis that high biopsychosocial case complexity would be related to more diagnostic interventions, this was not the case according to our differentiation of diagnostic interventions (see Table 5). Patient groups receiving standard examination (physical examination, lab, ECG) and more detailed examination at the ER (standard plus ultrasonic, X-ray, puncture, expert consultation, MRT and/or CT) did not differ in the INTERMED Score (F(2, 166) = 0.958, p = 0.386).

Forty patients (24%) were directly admitted after the ER visit, either to the Department of Internal Medicine or to other departments or external hospitals. Among all 40 patients admitted, data sets of those 32 patients who stayed in a department at the Medical University of Graz for subsequent inpatient treatment were further evaluated. A quarter of them (n = 8) received psychosocial interventions (consultation by a psychotherapist, psychiatrist and/or social worker). However, no recommendations for further psychosocial treatment after discharge were documented in the discharge letters.

Regarding all psychosocial interventions, both during the ER visit and subsequent inpatient treatment, 8% of all 167 patients received such interventions. In accordance with our hypothesis, biopsychosocial complexity seems to have contributed to the practice of these interventions. Twenty-one percent of cluster three received such interventions as compared to 7% of cluster one and 6% of cluster two.

## Discussion

### Case complexity in the emergency room and associated factors

This is the first study to explore biopsychosocial case complexity and associated complex health care needs in patients visiting an emergency room by using a validated interview method. According to our results, multidisciplinary health care needs seem to be a common phenomenon in the emergency room. They can be estimated to affect about 12–14% of patients in the investigated triage groups. It is widely accepted that psychosocial factors accompany the admission to an emergency room although they do not represent the primary focus of medical interventions [22], [25], [26]. However, our findings suggest that health care needs of these patients are seldom addressed in an adequate manner during the ER visit and subsequent inpatient treatment. In approximately 80% of highly complex patients, no psychosocial interventions were documented. In addition, in those 20% of patients receiving integrated treatment according to their complex health care needs, continuity of integrated care in outpatient facilities was rarely facilitated by appropriate recommendations in the discharge letters. Unfortunately, a discontinuity of care after discharge from hospital is associated with a high risk of adverse effects and adequate patient management would require improved communication between specialized and primary health care segments [27]. Such communication should also comprise information on psychosocial interventions and recommendations for further treatment.

In accordance with our hypotheses, highly complex patients seem to use this health care segment more often than less complex patient groups as reflected in the number of prior ER visits during the last year. This implies higher health care costs for complex patients [11]. It may be added that the number of ER visits is also known to be a risk factor for hospitalization [28].

Congruent with other study results [9], the highly complex patient group seems to be transferred for inpatient treatment most frequently. Yet, no significant difference regarding admission rates was found between these patients and patients with high somatic complaints but low psychosocial case complexity, as represented by cluster one. Thus, patients with a high biological INTERMED score were often admitted, reflecting the medical reality of an emergency room. However, the living situation – namely living alone – must be regarded as another specific trait of highly complex patients in our sample. Deficient social support as a well established health risk factor [29] may contribute to their overall health-related burden. This could be in line with the recent finding that social work in the ER might reduce the admission rate after an ER visit [30].

Interestingly, biopsychosocial case complexity does not seem to be reflected in the type and number of diagnostic procedures that are applied in the ER. According to our data, high case complexity was not related to more expensive diagnostic procedures at the ER.

### Strengths and Limitations

Although this is the first study that provides insight into the clinical handling of biopsychosocial complexity in the ER setting, several limitations need to be considered when interpreting our results. First of all, due to the study design excluding Manchester triage groups one and two, we cannot claim that these results are representative of all emergency room patients in internal medicine. Although this limitation may expand to the one week time span of data collection and to the small number of highly complex patients, in particular in the subgroup with inpatient treatment, the distribution of patients according to Manchester triage groups and the number of patients resembled the average patterns over the year. In addition, differences in national health care systems must be considered when generalising these data.

A team of 12 interviewers was necessary to guarantee an investigation over 7 days and 24 hours. In order to ensure reliability of the INTERMED ratings, training included several group ratings and double ratings of patients in internal medicine with one of the authors (W.S.) who has co-developed the INTERMED until each interviewer achieved official certification as an INTERMED rater. The INTERMED has been successfully applied in several studies suggesting reliability and validity concerning the measurement of biopsychosocial complexity. Nevertheless it does not allow for the detection of singular psychiatric diagnoses such as depression, anxiety or somatisation. Thus, patients with a high psychosocial but a low somatic burden will not reach the cut-off point for high biopsychosocial complexity and are not necessarily addressed by this study.

Finally, the application of a cluster analysis in addition to using a cut-off score could be questioned, as results by a cluster analysis need to be interpreted with much caution and are not easily transferable into clinical practice. Indeed, due to the exploratory character of this study, the cluster analysis was calculated in order to provide complementing insight into the whole spectrum of different levels of case complexity in an ER study cohort. In an ER setting in particular, patient groups with low biological and biopsychosocial complexity deserve to be detected and gain adequate attention in order to improve patient management overall. Thus, the results of the cluster analysis are reported to contribute to an understanding of the challenge of case complexity in the ER setting. Yet, these current results are exploratory and do not interfere with the established clinical recommendations for applying the INTERMED method in order to detect high biopsychosocial case complexity [31].

### Conclusions

Although biopsychosocial case complexity is a prevalent phenomenon in an emergency room, the corresponding health care needs are sparsely and inconsistently addressed in the ER and subsequent inpatient treatment. We therefore suggest that a screening system in the ER could help in the detection and treatment of this highly burdened group.

First, ER nurses could be easily trained in assessing high biopsychosocial complexity by applying the INTERMED interview. A screening of all patients who visit the ER for the 2^nd^ or 3^rd^ time within a year could be a time-efficient and standardized procedure to detect multidisciplinary healthcare needs among ‘frequent utilizers’ of this health care segment. Once detected, these patients should receive adequate psychosocial support as part of an integrated care plan. If they are dismissed, their family physicians should be informed and the relevant outpatient treatment facilities should be targeted for referral. If patients are referred for inpatient treatment, these complex care needs should be communicated to the departments that provide subsequent inpatient treatment. Specific psychosomatic inpatient facilities and CL-services seem appropriate to meet the biopsychosocial needs of highly complex patients. It could be argued that these suggestions imply additional health care expenditure. On the other hand, ER financial and staff capacities could be saved if treatment for the large group of patients with low scores in all domains (Cluster 2) was not provided in the ER but in less expensive and more adequate outpatient treatment facilities. Overall, detecting and managing the frequent challenge of biopsychosocial complexity in the emergency room setting could contribute to the ultimate goal of improving patient management and patient health outcomes as well as reducing health care expenditure. Interventional studies are urgently needed to demonstrate these effects.
